# Artificial Rupture of Membranes and Spontaneous Rupture of Membranes: A Systematic Review of Feto-Maternal Outcomes

**DOI:** 10.7759/cureus.77760

**Published:** 2025-01-21

**Authors:** Kalyani S Khodke, Nikita Vijay

**Affiliations:** 1 Obstetrics and Gynaecology, NKP Salve Institute of Medical Sciences and Research Centre, Nagpur, IND

**Keywords:** active phase of labour, amniotomy, artificial rupture of membranes, feto-maternal outcomes, spontaneous rupture of membranes

## Abstract

Artificial rupture of membranes (ARM), or amniotomy, is a frequently conducted technique for labor acceleration and dystocia prevention without adverse feto-maternal outcomes in women with spontaneous labor. However, in the literature, there has been controversy concerning the regular application of this technique. Therefore, the present systematic review was conducted to evaluate and compare the effect of ARM and spontaneous rupture of membrane (SRM) on feto-maternal outcomes. With the specified keywords, “spontaneous rupture of membrane,” “artificial rupture of membrane,” AND “active phase of labor” a literature search was conducted on ScienceDirect, Google Scholar, and PubMed databases between 2019 and 2024 for the inculcation of appropriate studies. Based on the eligibility criteria, five studies were included. The results revealed that the duration of labor was significantly reduced in the women with ARM compared to SRM, without an increment of the risk of fetal or maternal adverse outcomes. Hence, ARM can be a safe and cost-effective intervention in certain clinical scenarios, but selective rather than routine implementation may offer more balanced outcomes in labor management.

## Introduction and background

Intrapartum obstetric treatment is to minimize unfavorable consequences while delivering healthy infants to healthy mothers. This goal is accomplished during labor by closely monitoring the mother and the fetus and, if required, providing the necessary interventions. Amniotomy, or artificial rupture of membranes (ARM), is one of the labor-management treatments that was established to enhance labor without causing negative feto-maternal outcomes. With the main goals of labor acceleration and avoiding dystocia in women experiencing spontaneous labor, it is one of the most often used procedures in modern obstetrics. It occurs by purposeful rupture of the amniotic sac by the medical professional [1-3].

There have been several justifications for this deliberate rupture of the sac, some of which include the opportunity for more precise fetal status monitoring, the inducement and augmentation of labor, and the evaluation of the color of the amniotic fluid. The exact mechanism through which amniotomy expedites labor is yet unknown. It is believed that a rupture of the membranes causes a surge in prostaglandin and oxytocin synthesis and release, which intensifies contractions and accelerates cervical dilatation [4-7]. On the other hand, ARM may increase the risk of chorioamnionitis in rare cases, variable cord compression deceleration, and cord prolapse.

A number of obstetricians believed that amniotomies may expedite labor, but others believed that the benefits of ARM were overstated. In the 1930s, Eastman proposed that the "bag of water" around the fetus was essential for proper labor as it performs a crucial part of cervical dilation [5]. Many researchers agreed with the theory that pressure from the membranes that are intact contributed to the dilatation [8,9]. Furthermore, oxytocin surges are stimulated by the pressure applied by the membranes in a manner similar to that of the fetal presenting portion [10]. In the literature, there has been debate concerning the regular application of this technique. A notable decrease in the labor duration has been observed in certain trials, but no impact on other outcome measures was recorded [11,12]; however, other studies showed that the intervention had no discernible, consistent impact on the outcome measures, including labor duration [13]. Hence, the goal of the present systematic review was to evaluate and compare the effect of spontaneous rupture of membranes (SRM) and ARM on feto-maternal outcomes.

## Review

The current systematic review was conducted following the Preferred Reporting Items for Systematic Reviews and Meta-Analyses (PRISMA) guidelines [14].

Data sources and search strategy

By utilizing databases consisting of Google Scholar, ScienceDirect, and PubMed, a literature search was conducted with keywords including “artificial rupture of membrane”, “spontaneous rupture of membrane”, and “active phase of labor”.

Study screening and selection

For the screening process, studies elaborating on the feto-maternal outcomes of labor following ARM versus SRM, consisting of randomized controlled trials (RCTs) published between 2019 and 2024 in the English language and with full-text availability, were included for the review. Study designs other than those mentioned, studies with inappropriate information, with non-availability of full text, and not in the English language were excluded.

To confirm the inclusion of the studies, two reviewers independently evaluated each article. Initially, duplicate papers were removed by reviewing the titles and abstracts. Subsequently, further screening was done on the selected articles to exclude those that did not meet the qualifying criteria. Finally, the full text of the selected articles was screened in order to determine eligibility. Through consensus, any disputes or disagreements were resolved among the reviewers.

Data extraction

The reviewers independently gathered the data, which included the objectives of the studies, the research design, the sample size, the methodology, the results, the conclusion, and the quality evaluation. Before incorporation into the review, each study was assessed independently.

Quality assessment

The methodological quality was assessed using the third category of the Mixed Methods Appraisal Tool (MMAT), which provides grades as low, moderate, and high for quantitative RCTs. This tool is commonly used to analyze studies consisting of RCTs, qualitative, mixed techniques, cross-sectional, and non-randomized trials [15,16].

Data synthesis

A critical narrative technique involving figures, text, and tables was used as narrative synthesis to demonstrate the results of the studies [15]. Higher methodological quality study, potential biases, limitations, and other concerns throughout the data analysis provided a critical opinion for the evaluation. As the number of relevant research studies was limited, statistical synthesis or a meta-analysis was not performed.

Results

**Figure 1:**
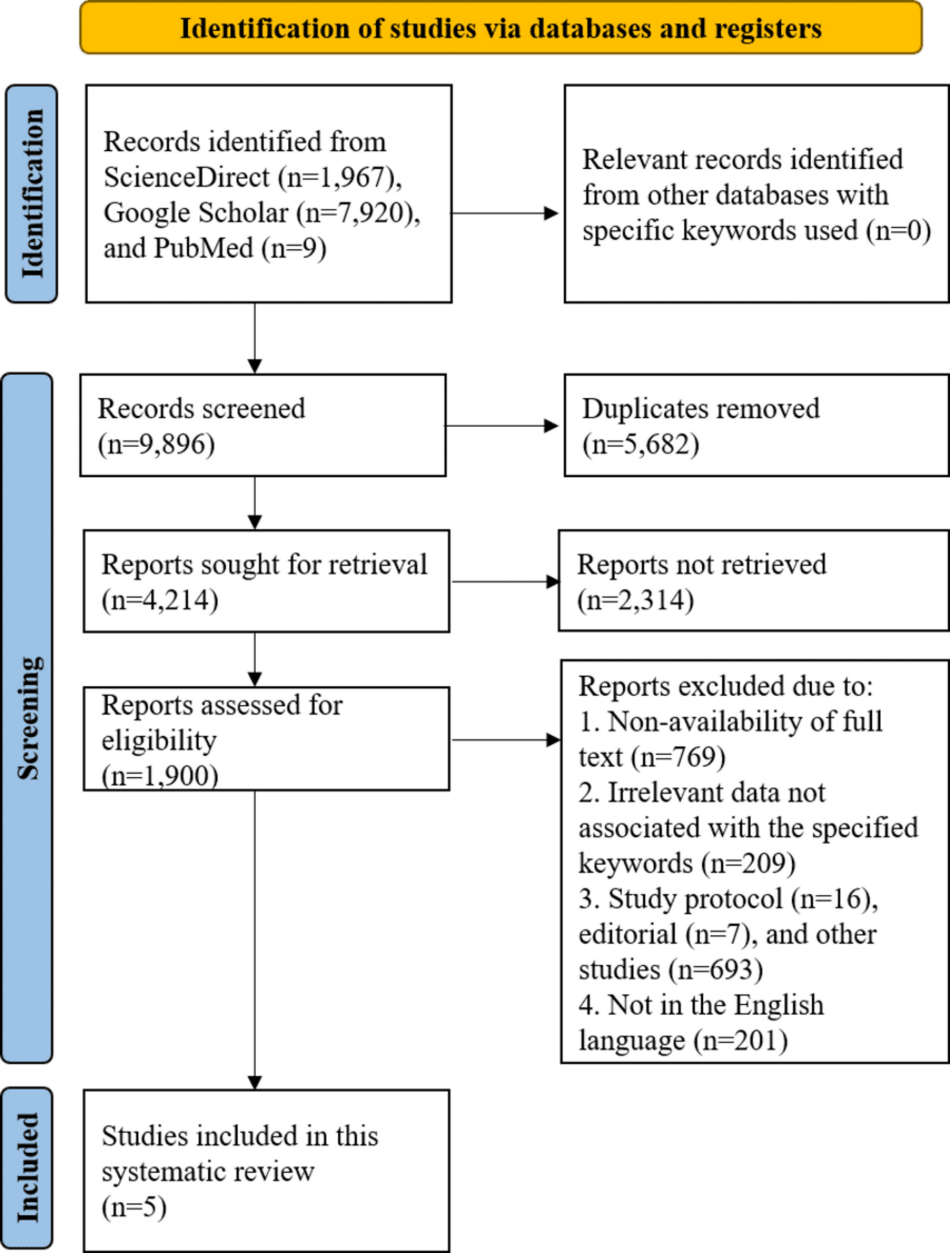
The PRISMA flowchart illustrating search strategy PRISMA: Preferred Reporting Items for Systematic Reviews and Meta-Analyses

Figure 1 presents the outline of the search strategy. In all, 9,896 studies were evaluated, which consisted of 7,920 articles from Google Scholar, 1,967 articles from ScienceDirect, and nine studies from PubMed. Following the removal of 5,682 duplicate publications, 4,214 articles underwent a retrieval examination, from which 2,314 were not recovered. Subsequently, the remaining 1,900 articles were screened for eligibility, of which 209 provided irrelevant data; non-availability of full text comprised 769 articles; other research studies involved 716 articles; and 201 studies were not presented in English and therefore were not included; hence, overall, five studies were included.

Extracted data from the included studies are summarized in Table 1.

**Table 1:** Extracted data from the included studies RCT: randomized controlled trial; ARM: artificial rupture of membranes; SRM: spontaneous rupture of membranes; PPH: postpartum hemorrhage; NICU: neonatal Intensive care unit; CS: cesarean section; APGAR: appearance, pulse, grimace, activity, and respiration

Sr. No.	Author and year	Aim and objectives	Study design	Sample size	Methodology	Results	Conclusion	Quality assessment
1.	Zandvakili S et al. (2019) [17]	To evaluate the effect of early ARM on labor indices and outcomes in nulliparous women in comparison to SRM.	RCT	146 (ARM = 73, SRM = 73)	Chorioamnionitis, the duration of the first and second stages of labor, APGAR scores at one and five minutes, dystocia rate, PPH, and extended labor	The first labor phase: ARM = 124.86 ± 51.41 and SRM = 187.12 ± 86.12 minutes; the second labor phase: ARM = 38.77 ± 15.27 and SRM = 44.62 ± 14.18 minutes; SRM = 10 prolonged labors; SRM = 2 cases of fetal distress; PPH: ARM = 2 cases (12.5%) and SRM = 14 cases (87.5%); APGAR score at first minute: ARM = 8.97 ± 0.12 and SRM = 8.71 ± 0.61; APGAR score at fifth minute: ARM = 9.98 ± 0.12 and SRM = 9.97 ± 0.19.	Early ARM resulted in a considerable reduction in the overall labor duration without a corresponding rise in the incidence of adverse complications for mothers and newborns.	High
2.	Subramaniyam B et al. (2020) [18]	To determine the efficacy of ARM in routine reduction of all spontaneously initiating uncomplicated labors and its impact on the feto-maternal outcomes as compared to the SRM group.	RCT	100 (ARM = 50, SRM = 50)	The second, third, and total duration of labor, active phase of dilatation, PPH, and APGAR scores were compared between the groups.	In the primi, second, and multigravida groups, a maximum number of participants showed three to six hours of the active phase of dilatation, which consisted of more participants in the ARM group in comparison to the SRM group. Additionally, the duration of the second stage of labor was between 0 to one/two hours, which consisted of more participants in the ARM group in the second and third gravid categories, whereas, in the primigravida group, the maximum number of participants were from the SRM group in comparison to the ARM group. Moreover, the majority of the participants across all three gravidae had a duration of the third stage of labor between 0-10 minutes, which for primigravida and second gravid categories had the maximum number of participants from the ARM group in comparison to the SRM group, and in the multigravida category, the majority of the participants were from the SRM group. Additionally, in the primigravida category, the total duration of labor observed among the majority of the participants was three to six hours in the ARM group and six to nine hours in the SRM group. However, in the second gravid category, in both groups, the duration was between three to six hours. Whereas in the third gravida category, the majority of the participants in the ARM group had a duration of labor between one to three hours and between three to six hours in the SRM group. Furthermore, findings of the APGAR score at one minute reported that in the primigravida, second gravida, and third gravida categories, the majority of the participants had having APGAR score of more than seven. However, the majority of the participants were in the ARM group in comparison to the SRM group in the primigravida and second gravida categories, whereas, in the third gravida category, the majority of the participants were from the SRM group. Moreover, PPH was observed more in the SRM group.	With the exception of the shorter overall labor duration in the ARM group, there was no variation in the results between the two groups.	High
3.	Chauhan A et al. (2020) [1]	To evaluate the labor outcome in patients with ARM and to compare it with the SRM group outcomes.	RCT	120 (ARM = 60, SRM = 60	In order to prevent cord prolapse, the head engagement was evaluated in the ARM group. Kocher's forceps were used to rupture membranes while adhering to all aseptic procedures. The liquor's color was observed, and pre- and post-surgery, the fetal heart rate was monitored. Labor was monitored, and patients with insufficient uterine contractions underwent oxytocin augmentation. The total labor duration was recorded, along with the mode of delivery. The birth weight, APGAR score, and requirement for NICU hospitalization were recorded after delivery. Moreover, comparable outcomes were also observed in the SRM group.	The mean duration of labor: SRM = 6.94±1.8 hours and ARM = 5.24±2.5 hours; mode of delivery (vaginal delivery) was almost similar in both groups. Additionally, there was no statistical difference in the APGAR score (<7) at one minute in both groups with ARM = two patients and SRM = one patient. Moreover, NICU admission: ARM = five babies for respiratory distress and SRM = four for jaundice.	ARM is an intervention that can be used to reduce labor duration without having a negative impact on the results for the mother and fetus.	High
4.	Pahwa A et al. (2021) [19]	To evaluate and compare the effects of ARM and SRM on feto-maternal outcomes	RCT	200 (ARM = 100, SRM = 100)	Fetal heart sounds were reviewed, and cord prolapse was ruled out immediately after the ARM before the finger was removed. Membranes in the SRM were allowed to spontaneously rupture. Fetal heart rate and partograms were used to track the progress of labor in both groups, along with intermittent auscultation. The labor duration, the indication of lower segment CS, the mode of delivery, the need for augmentation of pitocin, the Apgar score at the end of one and five minutes, and NICU admission were the major and secondary outcomes that were examined and compared across the groups.	The mean dilatation: ARM = 4.6 ± 0.49 cm and SRM = 4.7 ± 0.46 cm, oxytocin augmentation SRM>ARM, frequency of vaginal delivery: SRM = 89% and ARM = 90%; frequency of CS: ARM = 11% (non-progress = seven and fetal distress = four) and SRM = 10% (non-progress = five fetal distress = four, and cord prolapse = one). Birth weight of the neonates: ARM = 2.4 ± 0.35 kg and SRM = 2.5 ± 0.35 kg. There was no significant variation in APGAR scores and NICU admissions between both groups.	ARM is useful in shortening the duration of labor and lowering the need for oxytocin augmentation; it offers no additional benefits in terms of other outcomes for mothers and newborns. It may be more advantageous to perform a selective ARM as opposed to a routine one.	High
5.	Shafqat T et al. (2022) [20]	To compare the effects of ARM and SRM in primigravida on the labor duration.	RCT	80 (ARM = 40, SRM = 40)	With a 4 cm cervical dilatation and preventive antibacterial therapy, Kocker's forceps were used for ARM in an aseptic setting. In order to prevent placental abruption and cord prolapse, the liquid was allowed to drain. Liquor color was noted, and fetal heart sounds were recorded, and the progress of labor was evaluated by vaginal examinations performed every three hours. In the SRM group, patients were allowed to have spontaneous labor in which the membranes were intact for as long as feasible and were monitored.	The mean duration of labor: ARM = 5.7 hours and SRM = 7.15 hours.	In primigravida, ARM during active labor highlights a considerable decrease in the labor duration.	High

Discussion

In developing nations, the goal of improving intrapartum obstetric care through efficient obstetric services supports in reducing maternal and fetal morbidity and mortality rates. The objective of the current study was to compare the effects of ARM and SRM on labor and feto-maternal outcomes. Artificial rupture of membranes is a part of active labor management, which aims to lower the rate of dystocia, or cesarean sections (CS) for prolonged labor, although its impact is not found to be consistent with the labor duration. There is insufficient evidence to justify regular ARM for all women. It is recommended and regularly carried out for all women in certain centers, and it is utilized for women whose labors have become longer in many other centers. This illustrates the lack of agreement around the function of ARM.

The results of the present review highlighted that in all the studies reported, the mean duration of labor was higher in the SRM group, whereas the number of women was higher in the ARM group. Additionally, Zandvakili S et al. [17], Goffinct F et al. [21], and Fraser WD et al. [12] stated almost similar results. On the contrary, a recent systematic review [2] reported no correlation between ARM and reduction in the first stage of labor duration. The aforementioned finding is valid for both primiparous and multiparous subgroups; however, the outcome of the review may have been influenced by the high levels of heterogeneity across the included studies. Cammu H et al. [22] and Johnson N et al. [23] reported no significant findings of the first stage of labor after ARM in nulliparous pregnancies that are uncomplicated. The majority of studies indicate that ARM results in shorter labor duration. It is unclear if shorter labor has any therapeutic benefits, and it could compress all uterine work into a smaller amount of time, intensifying labor pain and increasing the risk of fetal heart rate anomalies. The drawbacks of iatrogenic intervention might outweigh this little benefit.

Additionally, the mean dilatation and augmentation of oxytocin were higher in the SRM group. The well-known impact of ARM on uterine contractions may help to explain this. Moreover, Smyth RM et al. [2], Fraser WD et al. [12], and Bellard MB et al. [10] correlated similar results. Moreover, postpartum hemorrhage (PPH), fetal distress, jaundice, non-progress, and cord prolapse were observed in the SRM group. Whereas, in the ARM group, non-progress and respiratory distress were observed. Additionally, Rasheed FA et al. noted that 6.7% of women had PPH in the SRM group and 14.3% in the ARM group [24]. Other studies done by Fraser WD et al. [12] and Bellard MB et al. [10] demonstrated that early ARM does not decrease or increase PPH.

The most common mode of delivery was vaginal in both groups; however, the ARM group consisted of more women in comparison to the SRM group. Additionally, the Apgar scores at one and five minutes of >seven and the mean birth weight were almost similar in both groups. Similar results were observed in the previous studies [2,10,12,12,21-23,25]. Additionally, the indications for CS were comparable among groups, and similar outcomes were reported in many studies [2,10]. Moreover, Goffinct F et al. [21] and Fraser WD et al. [12] reported that the majority of the CS was performed for fetal distress as either the only indication or as a contributing indication greater in the ARM than in the SRM group. This is because early detection of meconium reduces the threshold for earlier surgical delivery, and ARM leads to cardiotocographic abnormalities [19].

In summary, ARM proved to be more beneficial in comparison to SRM and significantly shortened the total duration of labor without an increment in the incidence of feto-maternal complications. Additionally, it may encourage the reduction of delivery units and staff, less monitoring time, and a shorter hospital stay.

Strengths and limitations

The studies reported the beneficial impact of ARM over SRM with the incorporation of high-quality studies. However, certain limitations were observed, which consist of the inclusion of limited studies due to the eligibility criteria imposed and no meta-analysis reporting due to the varying methodological section of the studies. Additionally, the studies published in other languages except English and in databases other than those considered were excluded, which may have limited the incorporation of valuable studies.

## Conclusions

This systematic review provides a comprehensive analysis of the impact of ARM versus SRM on feto-maternal outcomes during labor. The findings suggest that in comparison to the SRM, ARM significantly reduces the duration of labor without an increment in the risk of feto-maternal complications. The evidence indicates that ARM can be a cost-effective and safe intervention in certain clinical scenarios, but selective rather than routine implementation may offer more balanced outcomes in labor management. Furthermore, RCTs of high quality and with larger sample sizes are recommended to address the heterogeneity observed in existing studies and provide clearer guidance on the optimal use of ARM in different obstetric populations.
